# Permissive Summer Temperatures of the 2010 European West Nile Fever Upsurge

**DOI:** 10.1371/journal.pone.0056398

**Published:** 2013-02-19

**Authors:** Shlomit Paz, Dan Malkinson, Manfred S. Green, Gil Tsioni, Anna Papa, Kostas Danis, Anca Sirbu, Cornelia Ceianu, Krisztalovics Katalin, Emőke Ferenczi, Herve Zeller, Jan C. Semenza

**Affiliations:** 1 Department of Geography and Environmental Studies, University of Haifa, Haifa, Israel; 2 School of Public Health, University of Haifa, Haifa, Israel; 3 Department of Microbiology, Medical School, Aristotle University of Thessaloniki, Thessaloniki, Greece; 4 Hellenic Centre of Disease Prevention & Control, Athens, Greece; 5 National Center for Surveillance and Control of Communicable Diseases, Bucharest, Romania; 6 Cantacuzino National Institute for Research and Development in Microbiology and Immunology, Bucharest, Romania; 7 National Center for Epidemiology, Budapest, Hungary; 8 European Center for Disease Prevention and Control (ECDC), Stockholm, Sweden; Louisiana State University, United States of America

## Abstract

**Background:**

In the summer of 2010, Europe experienced outbreaks of West Nile Fever (WNF) in humans, which was preceded by hot spells. The objective of this study was to identify potential drivers of these outbreaks, such as spring and summer temperatures, relative humidity (RH), and precipitation.

**Methods:**

Pearson and lag correlations, binary and multinomial logistic regressions were used to assess the relationship between the climatic parameters and these outbreaks.

**Results:**

For human morbidity, significant (<0.05) positive correlations were observed between a number of WNF cases and temperature, with a geographic latitude gradient: northern (“colder”) countries displayed strong correlations with a lag of up to four weeks, in contrast to southern (“warmer”) countries, where the response was immediate. The correlations with RH were weaker, while the association with precipitation was not consistent. Horse morbidity started three weeks later than in humans where integrated surveillance was conducted, and no significant associations with temperature or RH were found for lags of 0 to 4 weeks.

**Conclusions:**

Significant temperature deviations during summer months might be considered environmental precursors of WNF outbreaks in humans, particularly at more northern latitudes. These insights can guide vector abatement strategies by health practitioners in areas at risk for persistent transmission cycles.

## Introduction

West Nile Virus (WNV) has circulated in Africa since at least 1937 and has been reported in the Middle East, India, Europe, and more recently in the New World since 1999. The Virus is transmitted among amplifying hosts, such as birds, and transferred by carriers, especially by *Culex* mosquitoes [1]. The enzootic cycle is driven by continuous Virus transmission to susceptible bird species through adult mosquito blood-meal feeding, which results in Virus amplification [2]. Certain mosquitoes can act as bridge vectors by biting both bird (reservoir) hosts and mammals and consequently transmit the Virus from the reservoir host to mammals [3]. Humans and horses, which are susceptible hosts, do not produce significant viraemia and are considered dead-end hosts [2], [4]–[9]. The basic transmission cycle of WNV occurs in rural and urban ecosystems. Both intrinsic biological barriers and extrinsic environmental determinants control persistent transmission cycles. The capacity of WNV to spread is a function of biotic (i.e., host abundance and diversity, host dispersal, such as long-distance avian migration, etc.) and abiotic (i.e., physical features of the environment and climate) factors. However, it is not clear whether the dispersal movement of migratory birds can explain the observed pattern of WNV spread [10]. As such, the presence of susceptible hosts, competent mosquitoes, and a pathogen are necessary but not sufficient for successful propagation of the transmission cycle. Rather, permissive meteorological conditions are a prerequisite for the persistence of active transmission. For instance, elevated temperatures positively affect the transmission cycle by increasing the rates mosquitoes bite their hosts per day, shortening the extrinsic incubation period of the Virus in the mosquitoes and also controlling survival rate of mosquitoes in the environment. For WNV specifically, high temperatures influence vector competence [11]–[14], accelerate Virus replication within mosquito vectors [6], [15], boost mosquito reproduction rates, and prolong their breeding season [15]–[18]. Epidemiologically, a number of outbreaks have been associated with favorable environmental temperature conditions for mosquito proliferation [19]–[22].

Over the last years, sporadic human cases or limited outbreaks of WNF have been reported in Europe [23]. However, during the summer of 2010, Europe and its neighboring countries experienced an unprecedented upsurge in the number of WNF cases in new areas, particularly in South Eastern Europe [24]. Moreover, numerous cases of WNV infection were reported concurrently in horses from several locations. These outbreaks of WNV infection in humans and horses coincided with extreme hot spells (according to the classification by NOAA [25]) during the preceding summer months, especially in Eurasia, where temperatures of more than 5°C above normal for the summer [26].

The climate in Europe and Eurasia is changing. According to the World Meteorological Organization, the warming trend peaked this past decade ending in 2010, which was also one of the top three warmest years ever recorded. During the summer of 2010, Eurasia endured exceptional heat waves while southeastern Europe experienced a record-setting sequence of consecutive hot nights [26]. We set out to examine whether some of the biotic and abiotic conditions of this 2010 outbreak could in part explain the upsurge in WNF and whether avian migration played a role in WNV dispersal.

## Materials and Methods

Data series of confirmed cases of WNF in humans reported from Russia (419 cases), Greece (262), Israel (104), Romania (56), Turkey (38), Hungary (16), Italy (7), and Spain (2) were compiled and analyzed. The WNF data was assembled by the ECDC from several sources [27]–[31]. The surveillance systems for the countries with sufficient WNF cases that lent themselves to statistical analysis are briefly described below.

The WNV surveillance system in Greece is part of the general surveillance system, whereby physicians in Greece notify the Hellenic Centre for Disease Control and Prevention (HCDCP) of all cases of WNV infection, using a somewhat modified 2008 European Union (EU) case definition [27], [29], [32]. A standardized reporting form is utilized to compile information regarding the demographic profile, clinical symptoms, underlying chronic medical conditions, potential risk factors, and laboratory results of the reported cases. Active surveillance is conducted through regular telephone inquiries to hospitals in the affected areas for case finding, follow-up and data validation. Moreover, in-depth telephone interviews are performed using a semistructured questionnaire to obtain a detailed exposure history of all cases. Cases reported as encephalitis (including meningoencephalitis), meningitis, or acute flaccid paralysis, are classified as West Nile neuroinvasive disease cases. All other cases are considered non-neuroinvasive.

The surveillance system in Romania for WNV neuroinvasive disease has been in place since 2009 for all the country’s districts [30]. Within the routine WNV surveillance activities in Romania, the 2008 European Union (EU) case definition is used for a suspected case with WNV infection [32]: any person over 15 years of age who presents with Fever and meningitis, encephalitis, or meningoencephalitis between May and October and who reports a history of mosquito bites. Two sets of samples are collected for each suspected case: for patients with acute symptoms both cerebrospinal fluid (CSF) and serum are taken. For patients in convalescence phase, a second serum sample is taken 14–21 days later. A probable or confirmed case of WNV infection is defined as a person who meets the relevant clinical and laboratory criteria for probable or confirmed cases described in the EU case definition. A suspected case is not considered if WNV-specific IgM was not detected in CSF and serum.

Since the epidemic in Israel in 2000, WNF has been included in the list of notifiable diseases; by law, each case must be reported to the Ministry of Health. The Ministry’s Central Virology Laboratory is responsible for standardizing the laboratory testing for WNF serology and serum samples, which are submitted to authorized serology laboratories. Any positive finding is reported to the Department of Epidemiology at the Ministry of Health, which collects the data in a central database [33] and regularly publishes them. Data were obtained from infectious disease hospitals and medical clinics and reported using a standardised form containing information on symptoms, onset date, and possible risk factors.

WNF cases in Russia are captured from hospitals, health care centres and the Centre for Hygiene and Epidemiology. Individuals that present with encephalitis, meningitis, or Fever without any specific diagnosis are tested for WNV-specific antibodies with an ELISA test (laboratory confirmed case defined as: IgM titer ≥1800) by the Regional Centre for Pathogenicity Group. Additionally, WNV RNA is detected by RT-PCR in blood serum, CSF or autopsy material as a supplementary diagnostic test. The national system for monitoring and laboratory diagnosis is composed of the Centre for Express Detection and Identification and the Reference Centre at the National level in Moscow. DNA sequencing for typing of the WNV lineage is carried out at the Central Research Institute for Epidemiology in Moscow. In 2010, passive surveillance in hospitals and health centres was complemented with active surveillance in all hospitals in the areas with active transmission of WNV. These epidemiologic investigations were supported with vector surveillance of mosquitoes in areas with and without WNV transmission [34].

In addition, confirmed data of WNV infection in horses during the spring-summer of 2010 reported from Italy (96 cases), Spain/Gibraltar (39), Greece (30), Morocco (25), Romania (5), and Bulgaria (5) were collected from the OIE (World Organization for Animal Health) website [35].

**Figure 1:**
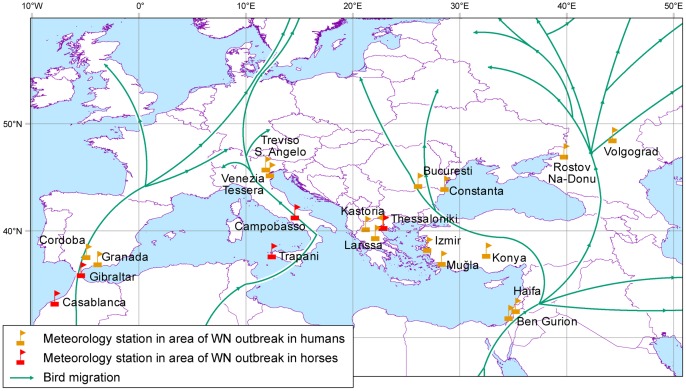
The meteorological stations at the main locations of WNV outbreaks in humans and horses, and a comparison with bird migration tracks over the study area in spring.

Sixteen formal WMO meteorological stations in each of the local sub-climates where the outbreak occurred in humans, as well as five meteorological stations for the equine morbidity sites, were selected (Figure 1). Most of the stations are located inside the outbreak district at a distance of less than 50 km from the area with the highest frequency of disease occurrence. Daily data of mean, minimum and maximum temperature, and relative humidity (RH) for each period beginning 1 March and ending 31 October over the 30 years from 1981 to 2010 (preceding and throughout the outbreak) were retrieved from the NCDC/NOAA Information Service [36].

Monthly Surface Gauss Precipitation Rate (Kg/mˆ2/s) for each meteorological station was collected from the Earth System Laboratory of NOAA. This dataset was used since the validation analysis found gaps in the daily rainfall data in the 30-year study period.

For each meteorological station:

To compare between specific dates within the 30-year study period, the daily data was averaged and combined into 35 artificial weeks of seven-day intervals [21]. Since WNV infections flare up in the summer and continue into the fall (CDC), our study period was selected to begin 1 March (prior to the outbreak) and end 31 October, with the first artificial week from 1 March to 7 March, and the last from 25 October to 31 October.

The mean weekly anomalies of the temperature/RH during the summer of 2010 compared with the perennial weekly average of 1981–2010 were calculated by normalizing the deviation to evaluate the anomaly of each factor: 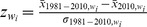, where *w_i_* indexes the *i^th^* artificial week mean values.

Pearson correlations were calculated between temperature/RH and WNV infection onset cases per dates for each concurrent week.

Lag-effects were explored, as it was assumed that the mosquito population abundance and Virus response may follow increasing ambient temperature/RH by several weeks [21]. Analyses were conducted for lags of 1 to 4 weeks of occurrence.

Binary Logistic Regression was used to assess whether there was any response to the temperature/RH deviations regardless of the specific number of weekly cases. This regression was used for predicting the outcome of a binary dependent variable based on one or more predictor variables. Thus, for each week, WNV infections were recorded as a binary response: either present or absent.

We used Multinomial Logistic Regression analysis to assess the relationship between temperature/RH deviations and the specific number of cases observed weekly. This model was used to predict the occurrence probabilities of the different possible outcomes of a categorically distributed dependent variable, given the independent variables. In our case the response variable (number of WNV infections/week) is a discrete random variable, and accordingly, the regression was used to analyze the properties of the above-mentioned data. Essentially, for each of the weather stations, two sets of five analyses were used to explore the statistical relationship between the variables and therefore, to control for family wise error rates (α, type I error). Accordingly, following the application of the Šidák correction [37], the initial significance level (α = 0.05) was set to α = 0.0102 as the test-specific rejection rate.

The same calculations above were computed for the precipitation datasets on a monthly basis.

## Results

### 1. WNF Cases in Humans

**Figure 2:**
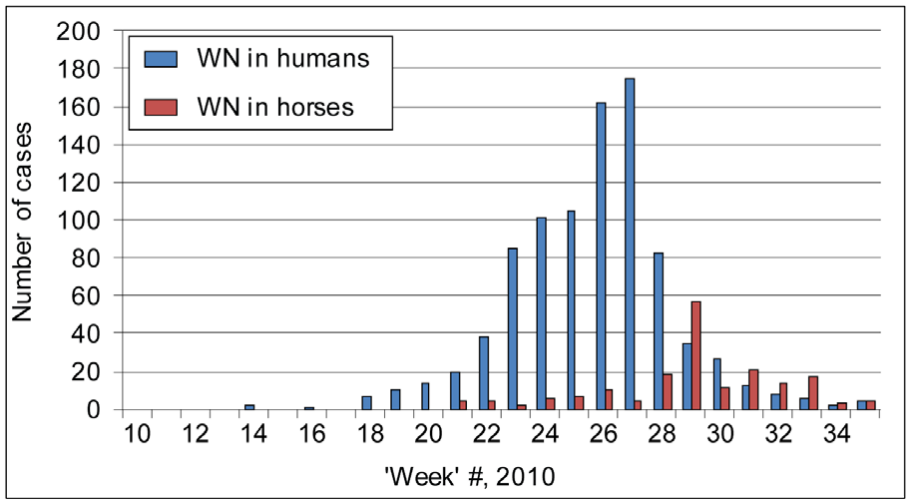
Cumulative numbers of reported WNV cases in humans and horses (all countries) during spring and summer 2010 (artificial week 10 is from 3 May to 9 May).

The first case of WNF in humans was reported in week 14 (31 May–6 June) in Israel. For all other locations, most of the cases were reported between 26 June and 31 October, while the main outbreak occurred between the end of July and the end of September. A clear peak was observed at weeks 26 and 27 (23 August–5 September) (Figure 2). The development of the eruption was characterized by one main peak in Greece, Russia, and Romania. The outbreak in Israel was different and characterized by a propagated spread perhaps as a result of a pest control policy.

#### 1.1. Temperature

**Figure 3:**
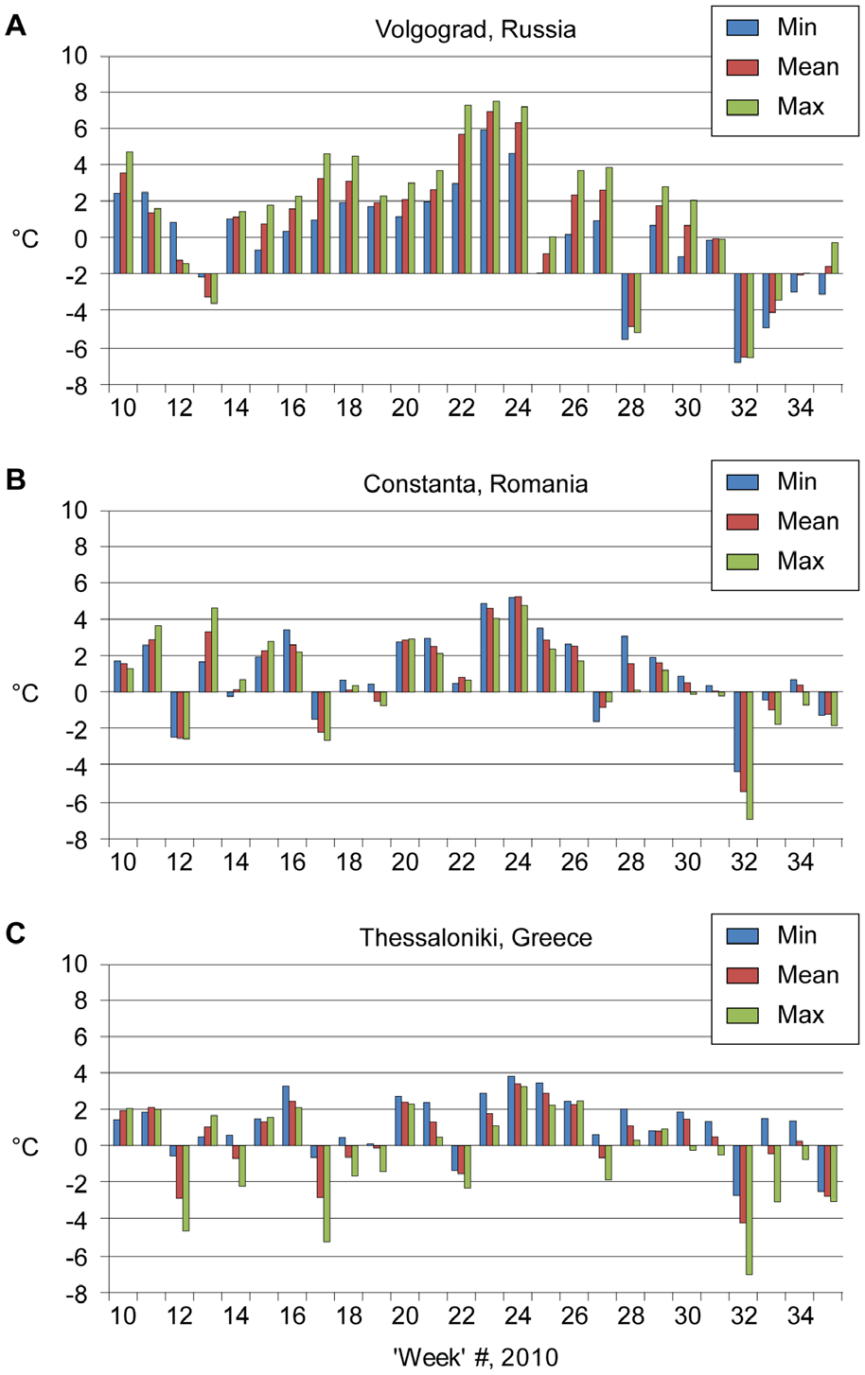
Weekly temperature anomalies (C°) during spring and summer 2010 for selected European stations (artificial week 10 precedes the disease upsurge by two months). Note: Deviation computed by subtracting weekly mean temperature of 2010 from the weekly perennial average temperature. A: Volgograd, Russia. B: Constanta, Romania. C: Thessaloniki, Greece.

Calculation of the weekly anomalies for each of the 2010 spring–summer temperature parameters clearly shows consistent positive anomalies (see examples in Figure 3). The period from the end of July to mid-August was extremely hot in Russia (deviations >9°C), in Romania and Turkey (>5°C), but less so in Greece (>3°C) and Israel (>4°C). A sharp decrease in the deviation from mean temperature occurred in week 32 (4 October–10 October) in all countries except in Israel. A different pattern was found in Hungary: June and part of July were very hot (>5°C), but the later period was colder than usual. In Italy, July was very hot, followed by more moderate months.

A comparison between Figures 2 and 3 revealed that the main WNF outbreak peak in humans appeared 2–3 weeks after the extreme temperature anomalies had occurred.

Pearson and lag correlations results (Table 1) are described on a meteorological stations scale. Significant and positive linkages (0.31≤r≤0.65) were detected between temperature and a number of WNF cases for the Russian stations at lags of 2–4 weeks but not for the concurrent week (lag 0). Positive correlations were also detected for Romania: in Bucharest at lags of 2–3 weeks and in Constanta at lags of 1–4 weeks (0.33≤r≤0.61). In general, the strongest lag time results were obtained for the northern countries (Russia and Romania). In contrast, in southern and hotter countries (Israel and Greece), the correlations appeared in the same week (lag 0) and presented an immediate response. At these stations, significance was also observed at longer lag-times of 1–2 weeks in Thessaloniki (Greece), for all lags in Larissa (Greece, for minimum and mean temperature), at lags of 1–3 weeks for Ben-Gurion Airport station (minimum temperatures), and for Haifa, Israel (mean temperature). Weak associations were found for the Turkish stations at lags of 3–4 weeks. A different pattern was detected for Budapest with significant results (0.32≤r≤0.50) at lags of 6–7 weeks. Correlations were not computed for Italy and Spain because of sample size constraints.

**Table 1:** Pearson and lag correlation coefficients (mean, minimum, and maximum) for weekly average temperatures and dates of WNF case onset at selected European/neighboring stations, 2010.

	Volgograd, Russia			Constanta, Romania		
Temperature	min	mean	max	min	mean	max
Lag 0	0.16	0.25	0.26	0.17	0.17	0.12
Lag 1 week	0.20	0.28	0.29	**0.38**	**0.39**	**0.33**
Lag 2 weeks	**0.38**	**0.44**	**0.44**	**0.59**	**0.61**	**0.56**
Lag 3 weeks	**0.63**	**0.63**	**0.61**	**0.52**	**0.55**	**0.51**
Lag 4 weeks	**0.61**	**0.65**	**0.64**	**0.37**	**0.40**	**0.38**
	**Thessaloniki, Greece**			**Ben Gurion Airport, Israel**		
**Temperature**	**min**	**mean**	**max**	**min**	**mean**	**max**
Lag 0	**0.49**	**0.37**	**0.31**	**0.35**	**0.36**	0.22
Lag 1 week	**0.45**	**0.36**	**0.32**	**0.33**	0.27	0.16
Lag 2 weeks	**0.37**	**0.30**	0.24	**0.34**	0.29	0.18
Lag 3 weeks	0.29	0.24	0.19	**0.37**	0.26	0.08
Lag 4 weeks	0.28	0.19	0.13	0.18	0.18	0.08

Note: Bold values = significant results at p<0.05.

Multinomial Logistic Regression and Binary Logistic Regression revealed significant relationships between the spring-summer temperatures and WNF cases for the stations in Russia, Romania, Greece, Turkey, and Israel – countries with the largest number of WNF cases (Table 2).

**Table 2:** Multinomial Logistic Regression results between summer temperatures and WNF cases for selected European/neighboring locations, 2010.

	Lag 0		Lag1		Lag2		Lag3		Lag4	
	p-value	est.	p-value	est.	p-value	est.	p-value	est.	p-value	est.
**Volgograd, Russia**										
min					**0.0098**	1.18	**0.0001**	2.51	**0.0002**	2.27
mean	0.018		0.0232	0.97	**0.0023**	**1.47**	**<.0001**	**2.69**	**<.0001**	2.84
max	0.09		0.0204	1.04	**0.002**	**1.58**	**0.0001**	**2.66**	**<.0001**	3.09
**Constanta, Romania**										
min	0.3067		0.1091		**0.0013**	2.13	**0.0021**	1.93	0.0362	1.04
mean			**0.007**	12.05	**0.0007**	2.20	**0.0017**	1.85	0.03	1.03
max					**0.0019**	2.02	**0.0043**	1.76	0.028	1.19
**Larissa, Greece**										
min	**0.0064**	2.00			0.259	1.47	0.0325	1.41	0.0395	1.36
mean			0.0253	1.28						
max										
**Ben Gurion Airport, Israel**										
min	0.0225	1.51	0.0123	1.7	0.0106	1.74	0.0179	1.58		
mean	**0.0064**	1.46			0.0331	1.10				
max										

Note: P-value of all results <0.05. Bold results are significant according to the Šidák correction (α critical = 0.01021).

While in Rostov, Volgograd (Russia), and Constanta (Romania) significant correlations were found for all independent variables; in Bucharest (Romania) they were detected for the minimal and mean temperature. At Larissa (Greece), significant correlations were observed for the minimum temperature deviations, and at the Israeli stations, results were significant for the mean temperatures.

**Figure 4:**
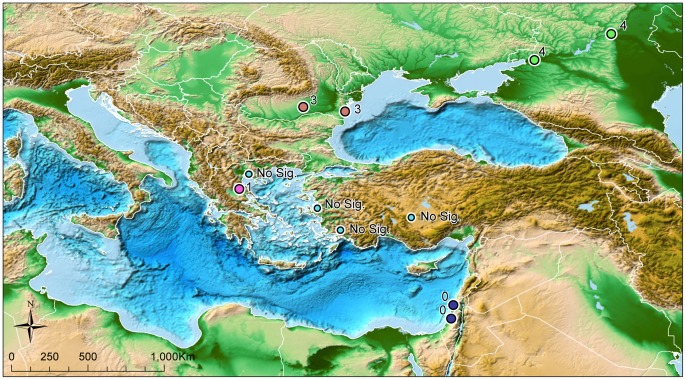
The maximal significant lag-time observed at each station between temperature deviations and emergence of WNF in humans.

The results displayed a geographic latitude gradient (Figure 4, the basic map source is [38]): in the northernmost stations, significant correlations between WNF cases and temperature anomalies were associated with a lag of up to four weeks after instances of increased temperatures in contrast to the southern stations that exhibited an immediate response, where the lag was much shorter or non-existent. At northern stations, significance was also observed at shorter lag-times, but the correlations became more apparent and stronger as the lag-time increased. For example, at the Volgograd station, a significant value of p = 0.002 was observed between number of WNF cases and maximum ambient temperatures two weeks prior to the occurrence of cases. The significance of the multinomial regression analysis with the maximum temperatures four weeks prior to observed WNF cases was p<0.0001.

#### 1.2. Relative humidity (RH)

Calculation of weekly anomalies of spring–summer 2010 for the RH clearly shows that in most stations the RH follows the perennial averages with a slight deviation. Considerable negative anomalies were found for Russia and less so for Turkey. In Hungary, positive anomalies were detected.

Pearson and lag correlations between the RH and the dates of WNF onset yielded significant results only in five stations - Rostov and Volgograd (Russia), Constanta (Romania), Izmir (Turkey), and Haifa (Israel). Negative correlations were found for the Russian stations at all lags (−0.34>r>−0.64) with increasing linkage paralleling the lag time. Negative weak/medium results were detected for Constanta at lags 0–1 and for Haifa at lags 1–4. Since an increase in the temperature value leads to a decrease in the relative humidity, the linkage between the negative association between RH and the WNF cases is reasonable. Multinomial Logistic Regression and Binary Logistic Regression were significant only for the Russian stations. Thus, the importance of RH as an indicator for WNF emergence is weak compared with the temperature values.

#### 1.3. Precipitation

The precipitation patterns were analyzed in relationship to the 2010 WNF outbreaks in humans. For example, in Volgograd (Russia), the precipitation was above perennial averages in May, but dry conditions existed during the rest of the summer. In Constanta (Romania), a clear peak was found in June, while other months were characterized by below-average rainfall amounts. The rainfall in June may explain the significant correlation with the lag of about eight weeks found for this station. In Thessaloniki, a slight increase in rainfall amounts was found during the summer months.

### 2. WNV Infection in Horses

The earliest cases of WNV infection in horses were reported in week 21 (19 July–25 July) in Western Morocco, the most southwestern site, where surveillance is routinely conducted. Subsequently, WNV infection in horses appeared in northeastern locations such as Greece, where intermittent surveillance is triggered by human cases, but also in Spain [39] and southern Italy, where surveillance in horses was carried out. Most of the cases were reported between 16 August and 31 October (week 25–35), while the main outbreak occurred between 6 September and 17 October, with a main peak in week 29 (13–19 September) in Italy, where 56 confirmed cases were reported in one week. It is important to note that during 2010, the equine outbreaks period started three weeks later than the WNF upsurge in human populations, where integrated surveillance was conducted (Figure 2).

#### 2.1. Temperature

Calculation of the weekly anomalies of summer 2010 again clearly shows consistent positive anomalies of the air temperature during this summer. For example, in Casablanca (Morocco), warmer conditions were observed for nearly the whole period (≤5°C). A similar pattern was found for Gibraltar but with lower anomalies.

In Trapany (Sicily, Italy), the whole summer was very warm with mean anomalies of up to 5°C. The temperature behavior in Campobasso (Molise, Italy) was characterized by different patterns with inconsistent fluctuations of extreme heat (6°C) for some of the weeks and below-average conditions in others (negative anomaly of more than 6°C).

Pearson and lag correlations between the weekly temperatures and the dates of WNV infection onset in horses did not reveal significant results at lags of 0–4 weeks. However, correlations were detected for longer periods of 5–10 weeks for Gibraltar, Trapany, Campobasso, and Thessaloniki.

Adjusting the α-error rate to account for multiple comparisons yielded that, in fact, significant results were obtained only for Trapany. This may be a result of the extreme increase in the number of cases - 56 in one week in this area, which imposed a strong leverage on the correlation analysis. Multinomial Logistic Regression and Binary Logistic Regression between the temperature and the equine morbidity dates did not reveal significant results. It is important to note that these findings are different from our results for the WNF in humans that showed a significant association between temperatures and morbidity at 0–4 weeks with a consistent geographic latitude gradient.

#### 2.2. Relative humidity (RH)

RH calculations of weekly anomalies during the spring and summer in 2010 clearly show that in most stations they follow the perennial averages with a slight deviation. Considerable positive anomalies were found only for Campobasso.

Pearson and lag correlations between the RH and the dates of WNV infection onset found weak to medium negative significant results for Trapany (Italy) at a lag of 5 weeks (r = −0.41) and for Thessaloniki at lags of 1 and 4–6 weeks (−0.30>r>−0.39). Multinomial Logistic Regression and Binary Logistic Regression between the RH and the equine morbidity dates were non-significant.

#### 2.3. Precipitation

The analysis could not point to a clear linkage between rainfall amounts and WNV infection onset in horses. However, a comparison between the precipitation rates of March–September 2010 and the mean rates of the 30-year period show that Trapany, Campobasso, and Thessaloniki were rainier than usual in July. These precipitations might have increased the standing water availability, an important breeding resource for mosquitoes. The rainfall increase in Gibraltar during August may explain the delayed appearance of the disease observed in this area.

### 3. Bird Migration Tracks

Figure 1 shows a comparison between the bird migration tracks during spring (over the Middle East, the Mediterranean, Europe, and Asia, [40]) and WNV infection outbreak locations. The linkage between the migration tracks and the outbreak areas is very clear in Israel, Turkey, Romania, Russia, Italy, and Spain (human cases) and in Morocco, Gibraltar, and Italy for equine morbidity. Although the linkage is less apparent for Greece, its location between two main pathways may be significant but requires further research.

## Discussion

The epidemiology of WNV transmission is controlled by a number of drivers. One of these is environmental temperature, which has been associated with a number of outbreaks [19]–[21], [41]. Temperature determines vector competence for arboViruses in general, and WNV in particular [11]. Elevated temperatures accelerate mosquito development as well as viral infection, dissemination, and transmission through increased Virus replication within the mosquito [42]. Thus, rapid amplification of both mosquitoes and Virus particles directly affects the likelihood of mosquitoes reaching maturity and subsequently infecting other hosts [6], [15]. Moreover, elevated ambient temperatures boost mosquitoes reproduction rates, the number of blood meals, prolongs the mosquito breeding season, and shortens the maturation period for the microbes they disperse [6], [15], [43]. For example, it has been proven experimentally that high temperatures during the incubation period affects WNV transmission of Cx. pipiens and profoundly influences mosquito-to-vertebrate transmission rates [16], [44].

Indeed, uncharacteristically elevated temperatures during the summer of 2010 were correlated with the WNF upsurge in humans, particularly in southeastern Europe, where newly affected areas were identified. We found a geographic latitude gradient: “colder” countries of more northern latitude (Russia and Romania) displayed strong statistically significant correlations between WNF cases and temperature anomalies with lags of up to four weeks from the onset of increased temperatures. In contrast, “warmer” and more southern countries (Israel and Greece), presented correlations with a minimal delay. These observations are in agreement with the notion that the force of WNV transmission is reduced at the confines of its northern latitude where the transmission cycle is constrained by extrinsic environmental conditions. However, once the threshold temperature limits are surpassed, we observe strong correlations between WNF outbreaks and conducive summer temperatures, particularly in northern-latitude countries that are latently hospitable to the spread of WNF. Permissive summer temperatures give rise to persistent WNF transmission cycles only under favorable and converging abiotic and biotic conditions. Temperature is one of these disparate factors that interact in complex and synergistic ways that has given rise to a number of WNF outbreaks in different settings [23],[45],[46]. The recurrence of elevated ambient temperatures, more frequently occurring and intense climate-change scenarios, and enduring heat waves might thus lead to even more WNF outbreaks, also in higher-latitude countries [47]. Although the data on WNF in Europe is limited to a short study period, our results are consistent with a number of studies that showed a clear association between extreme heat and outbreak intensity [6], [11], [15], [16], [20]. Moreover, recent events in Israel and New York City illustrate that even if the summer subsequent to the first eruption that coincided with extreme heat is less hot, the disease becomes endemic in the region [21], [48].

The associations with WNF outbreaks and relative humidity were weaker in our analyses, while the association with precipitation was not consistent across different outbreak locales. The interplay of these different abiotic factors is complex and calls for high-resolution field investigations to account for all the variables. Precipitation does not uniformly impact WNV transmission and may involve different Virus strains, vector mosquitoes, and human infrastructure [46], [49], [50]. Indeed, our inconsistent findings regarding the precipitation patterns reflect some of the conflicting observations documented in the peer-reviewed literature that deals with the complex impact of this factor with regard to WNF: on one hand, heavy rainfall during spring may increase standing water resources at the beginning of the hot season. In contrast, during drought conditions, standing water pools become richer in the organic material that mosquitoes need to thrive. This may encourage birds to circulate around small water holes and thus increase the interactions with mosquitoes [6], [15], [48], [51]. A very recent example is the WNF outbreak in Texas, USA in the summer of 2012. This outbreak was attributed in part to a drought conditions, which has reduced water flow and created stagnant water pools ideal for breeding mosquitoes [52].

The location of the horse- and human-WNV occurrences did not coincide (except in Thessaloniki) and equine morbidity started three weeks later than in humans. Significant associations between temperature/RH and WNV in horses were not found for lags of 0–4 weeks, contrary to the results in humans. Associations were found for longer lags in southern Italy. Summer rainfall in July and/or August might contribute to the eruptions at the end of the summer. Moreover, with the exception of Italy, which also conducts national surveillance, only a few sporadic equine cases that were reported cannot be classified as true outbreaks of WNV infection. Surveillance in horses and humans is still inconsistent throughout Europe and thus data cannot be compared across countries; some countries experienced cases but did not report them.

The ability of WNV to spread quickly along migration routes has been demonstrated for North America. The precise mechanism of the Virus spread by migrating birds is still unknown and requires further research [10], [53], [54]. However, the clear linkage seen between the main migration tracks over Europe and Western Asia and the outbreak areas in our research may contribute to predictions of disease spread in Europe and neighboring countries.

Although it was beyond the scope of this study to examine other transmission drivers such as socioeconomic (e.g., human migration) and environmental (e.g., land use, vector life history traits, etc.) factors, it is known that they also played a role in this outbreak. However, environmental temperature should be considered a major factor in the risk assessment of WNF outbreaks in the coming seasons. Since the warming is a continuing trend, it should be taken into account when evaluating the risk of WNV transmission in Europe in coming years.
